# Supplementary material for: Utilization of health insurance by patients with diabetes or hypertension in urban hospitals in Mbarara, Uganda
Source: PLOS Glob Public Health. 2023 Jun 14;3(6):e0000501. doi: 10.1371/journal.pgph.0000501 (PMC10266657; doi:10.1371/journal.pgph.0000501)
Supplement: Attachment — Submitted filename: Response to Reviewers.docx [file pgph.0000501.s001.docx]

Dear Editor,
Thank you for reviewing our manuscript PGPH-D-22-00660 “Utilization of health insurance by patients with diabetes or hypertension in urban hospitals in Mbarara, Uganda”. We have found the time and carefully revised the manuscript and addressed the points raised during the review process. A marked-up copy of the manuscript labeled 'Revised Manuscript with Track Changes' and an unmarked version of the revised paper without tracked changes labeled 'Manuscript' have been uploaded as required.

We thank you and the reviewers again for all the suggestions which have greatly improved the manuscript.

Best regards,
Dr. Peter Kangwagye

Corresponding Author

Email: peterkangwagye@gmail.com

**Journal Requirements:**

1. Please amend your detailed Financial Disclosure statement. This is published with the article. It must therefore be completed in full sentences and contain the exact wording you wish to be published.

a. Please clarify all sources of funding (financial or material support) for your study. List the grants (with grant number) or organizations (with url) that supported your study, including funding received from your institution. 
b. State the initials, alongside each funding source, of each author to receive each grant.
c. State what role the funders took in the study. If the funders had no role in your study, please state: “The funders had no role in study design, data collection and analysis, decision to publish, or preparation of the manuscript.”
d. If any authors received a salary from any of your funders, please state which authors and which funders.

If you did not receive any funding for this study, please simply state: “The authors received no specific funding for this work.”

***Response****: We did not receive any funding for this study and have thus stated: “The authors received no specific funding for this work.”*

2. In the online submission form, you indicated that "Data is available via peterkangwagye@gmail,com.”. All PLOS journals now require all data underlying the findings described in their manuscript to be freely available to other researchers, either 1. In a public repository, 2. Within the manuscript itself, or 3. Uploaded as supplementary information.
This policy applies to all data except where public deposition would breach compliance with the protocol approved by your research ethics board. If your data cannot be made publicly available for ethical or legal reasons (e.g., public availability would compromise patient privacy), please explain your reasons by return email and your exemption request will be escalated to the editor for approval. Your exemption request will be handled independently and will not hold up the peer review process, but will need to be resolved should your manuscript be accepted for publication. One of the Editorial team will then be in touch if there are any issues.

***Response****: We have checked our raw data and found that the necessary data is already provided within the manuscript. We have thus revised the statement to “All the necessary data is provided within the manuscript”.*

**Additional Editor Comments (if provided):**
Please respond the reviewers' comments point by point and do necessary revisions.
Reviewers' comments:
Reviewer's Responses to Questions

**Comments to the Author**
1. Does this manuscript meet PLOS Global Public Health’s [publication criteria](https://journals.plos.org/globalpublichealth/s/criteria-for-publication)? Is the manuscript technically sound, and do the data support the conclusions? The manuscript must describe methodologically and ethically rigorous research with conclusions that are appropriately drawn based on the data presented.

Reviewer #1: Yes

Reviewer #2: Yes

***Response****: We are grateful to the two reviewers for finding the time to review this manuscript. We are glad that both agree that the manuscript meets PLOS Global Public Health’s* [*publication criteria*](https://journals.plos.org/globalpublichealth/s/criteria-for-publication)*. Thanks.*

2. Has the statistical analysis been performed appropriately and rigorously?

Reviewer #1: I don't know

Reviewer #2: Yes

***Response****: Although Reviewer#1 did not explicitly indicate that the statistical analysis had been performed appropriately and rigorously, we are confident that our work fully meets this criterion. Our confidence stems from the fact that the study was subjected to rigorous checks by an experienced team of co-authors and by faculty and external examiners at Bishop Stuart University.*

3. Have the authors made all data underlying the findings in their manuscript fully available (please refer to the Data Availability Statement at the start of the manuscript PDF file)?
The [PLOS Data policy](https://journals.plos.org/globalpublichealth/s/data-availability) requires authors to make all data underlying the findings described in their manuscript fully available without restriction, with rare exception. The data should be provided as part of the manuscript or its supporting information, or deposited to a public repository. For example, in addition to summary statistics, the data points behind means, medians and variance measures should be available. If there are restrictions on publicly sharing data—e.g. participant privacy or use of data from a third party—those must be specified.

Reviewer #1: Yes

Reviewer #2: No

***Response****: All the necessary data is already provided within the manuscript.*

4. Is the manuscript presented in an intelligible fashion and written in standard English?
PLOS Global Public Health does not copyedit accepted manuscripts, so the language in submitted articles must be clear, correct, and unambiguous. Any typographical or grammatical errors should be corrected at revision, so please note any specific errors here.

Reviewer #1: Yes

Reviewer #2: Yes

***Response****: Thanks.*

5. Review Comments to the Author
Please use the space provided to explain your answers to the questions above. You may also include additional comments for the author, including concerns about dual publication, research ethics, or publication ethics. (Please upload your review as an attachment if it exceeds 20,000 characters)

Reviewer #1: Excellent and important effort. Introduction lacks definition and further clarity in regard to microfinance operation. Authors need to take a more thorough look into the process as an outsider. Fixing the definition is key.

***Response****: Thanks for the comment. We have revised the manuscript and provided more details in regard to microfinance operation.*

Reviewer #2: The authors have reported the utilization of health insurance among a cohort of treatment-seeking patients with diabetes and hypertension in two hospitals in a region of Uganda. The article addresses a pertinent subject of global health importance but needs improvement to improve readability and reproducibility.

***Response****: Thanks for the time and care accorded to our manuscript. We have revised the manuscript and tried as much as possible to address the concerns raised.*
Methods
1. Is the questionnaire interviewer-administered or self-administered. Please state

***Response****: The questionnaire was administered by interviewers. We have clarified this in the revised the manuscript.*
Results
2. The response rate of the respondents?

***Response****: The questionnaire was administered to 370 participants after approaching 387 patients, with a response rate of 96%. These details have been added as required.*

3. Define abbreviations at first mention E.g UAP in line 170

***Response****: We have checked the abbreviations used and made the suggested revision. UAP is not our abbreviation but a company name which we now indicate in full as UAP Old Mutual Uganda Insurance.*
4. Line 208-209 is a repetition of the methods

***Response****: Line 208-209 has been deleted.*
5. When reporting results, the frequencies and then % of the discrete variables should be presented.
***Response****: We prefer the approach where we provide frequencies and then % of the discrete variables in the first paragraph of the Results section and thereafter put more emphasis on the percentage of respondents involved. This avoids having to repeatedly give figures which are already provided in tables.*

6. The tables are not well laid out. They need reworking to allow the rows and columns to align properly
***Response****: We have reworked the tables to allow rows and columns to align properly.*

7. Stick with a single appropriate measure of central tendency rather than reporting mean, median and mode.

***Response****: We have opted to report the mean as the main measure of central tendency. Median values have been deleted.*
8. Add legends to abbreviations (NHIS, OOP e.t.c) below the tables
***Response****: We have added the abbreviations in Table legends.*

9. Report likelihood in ratios rather than percentages
***Response****: We feel that percentages are easier for many readers to interpret the results. We have provided odds ratios in brackets so as to cater for those who prefer the latter.*

Discussion
The discussion could be improved by exploring the models of micro-health finance schemes in Uganda and how that could enhance service delivery and uptake of policies.
(Aranas LL, Khanam R, Rahman MM, Nghiem S. Combining Microfinance and Health in Reducing Poverty-Driven Healthcare Costs: Evidence From the Philippines. Front Public Health. 2020 Oct 8;8:583455. doi: 10.3389/fpubh.2020.583455. PMID: 33134241; PMCID: PMC7578378.)

Another limitation is that the study method might not be sufficient to explore factors that could influence use of health insurance schemes. The themes generated form the open ended questionnaire could form the basis for a qualitative study to explore other factors this study could have missed.

***Response****: We have incorporated both suggestions and feel they have helped improve our work. Thanks.*

6. PLOS authors have the option to publish the peer review history of their article ([what does this mean?](https://journals.plos.org/globalpublichealth/s/editorial-and-peer-review-process#loc-peer-review-history)). If published, this will include your full peer review and any attached files.
**Do you want your identity to be public for this peer review?** If you choose “no”, your identity will remain anonymous but your review may still be made public.
For information about this choice, including consent withdrawal, please see our [Privacy Policy](https://www.plos.org/privacy-policy).

Reviewer #1: **Yes:** Ghassan K. Abou-Alfa

Reviewer #2: No

[NOTE: If reviewer comments were submitted as an attachment file, they will be attached to this email and accessible via the submission site. Please log into your account, locate the manuscript record, and check for the action link "View Attachments". If this link does not appear, there are no attachment files.]
While revising your submission, please upload your figure files to the Preflight Analysis and Conversion Engine (PACE) digital diagnostic tool, <https://pacev2.apexcovantage.com/>. PACE helps ensure that figures meet PLOS requirements. To use PACE, you must first register as a user. Registration is free. Then, login and navigate to the UPLOAD tab, where you will find detailed instructions on how to use the tool. If you encounter any issues or have any questions when using PACE, please email PLOS at [figures@plos.org](mailto:figures@plos.org). Please note that Supporting Information files do not need this step.
