# Supplementary material for: Utilization of health insurance by patients with diabetes or hypertension in urban hospitals in Mbarara, Uganda
Source: PLOS Glob Public Health. 2023 Jun 14;3(6):e0000501. doi: 10.1371/journal.pgph.0000501 (PMC10266657; doi:10.1371/journal.pgph.0000501)
Supplement: Attachment — Submitted filename: Response to reviewers.docx [file pgph.0000501.s002.docx]

Dear Editor,

Thank you for reviewing our manuscript PGPH-D-22-00660R1 “Utilization of health insurance by patients with diabetes or hypertension in urban hospitals in Mbarara, Uganda”. We have revised the manuscript and carefully addressed the concerns raised. A marked-up copy of the manuscript labeled 'Revised Manuscript with Track Changes' and an unmarked version of the revised paper without tracked changes labeled 'Manuscript' have been uploaded as required.

We are very grateful to you and the two reviewers for the comments and hope and pray that you find the manuscript ready for publication in PLOS Global Public Health.

Best regards,

Dr. Peter Kangwagye

Corresponding Author

Email: peterkangwagye@gmail.com

**Comments to the Author**
1. If the authors have adequately addressed your comments raised in a previous round of review and you feel that this manuscript is now acceptable for publication, you may indicate that here to bypass the “Comments to the Author” section, enter your conflict of interest statement in the “Confidential to Editor” section, and submit your "Accept" recommendation.

Reviewer #1: All comments have been addressed

Reviewer #3: (No Response)

***Response****: We thank the two reviewers for their time and comments.*

2. Does this manuscript meet PLOS Global Public Health’s [publication criteria](https://journals.plos.org/globalpublichealth/s/criteria-for-publication)? Is the manuscript technically sound, and do the data support the conclusions? The manuscript must describe methodologically and ethically rigorous research with conclusions that are appropriately drawn based on the data presented.

Reviewer #1: Yes

Reviewer #3: Partly

***Response****: While Reviewer #3 understandably has concerns regarding methodological aspects of the study and analyses, we think this was due to our weak attempt to include qualitative and quantitative methods in our research approach. As can be readily judged, our study primarily used a quantitative approach. We have thus revised and left out the aspects referring to qualitative data.*

3. Has the statistical analysis been performed appropriately and rigorously?

Reviewer #1: Yes

Reviewer #3: Yes

***Response****: Thanks for the comments.*

4. Have the authors made all data underlying the findings in their manuscript fully available (please refer to the Data Availability Statement at the start of the manuscript PDF file)?

The [PLOS Data policy](https://journals.plos.org/globalpublichealth/s/data-availability) requires authors to make all data underlying the findings described in their manuscript fully available without restriction, with rare exception. The data should be provided as part of the manuscript or its supporting information, or deposited to a public repository. For example, in addition to summary statistics, the data points behind means, medians and variance measures should be available. If there are restrictions on publicly sharing data—e.g. participant privacy or use of data from a third party—those must be specified.

Reviewer #1: Yes

Reviewer #3: No

***Response****: We feel that we have provided the necessary data and that the concerns raised by Reviewer #3 could be due to our limited use of qualitative data. We have revised the manuscript and left out aspects regarding the qualitative approach since our study primarily used a quantitative approach.*

5. Is the manuscript presented in an intelligible fashion and written in standard English?

PLOS Global Public Health does not copyedit accepted manuscripts, so the language in submitted articles must be clear, correct, and unambiguous. Any typographical or grammatical errors should be corrected at revision, so please note any specific errors here.

Reviewer #1: Yes

Reviewer #3: No

***Response****: We are not sure about the specific error(s) spotted by Reviewer #3. Could this be about the limited use of qualitative data? We welcome specific suggestions for improvement.*

6. Review Comments to the Author

Please use the space provided to explain your answers to the questions above. You may also include additional comments for the author, including concerns about dual publication, research ethics, or publication ethics. (Please upload your review as an attachment if it exceeds 20,000 characters)

Reviewer #1: Thanks for the authors for the thorough review. No further comments

Reviewer #3: Dear Author
Thank you for coming up with the revised manuscript. The abstract, introduction, write up, quantitative inferencing were found in a scientific way. Although you have mentioned in the "data management and analysis" about analysis of qualitative data, this part was not found so well elaborated in the manuscript and needs to be further rectified. The following are recommended :
1. The tool is mentioned as both for 'quanti' and 'quali' but no detail given about the open-ended/quali part. Kindly describe about the quali-tool under tool subheading. May be the tool be needed to submit under supplemental file.
2. Regarding sample for qualitative part not mentioned elsewhere. Pls add along with qtt samples, number of respondents for qualitative information, you included in the study.
3. For data analysis, also submit the codes, sub-themes, themes in a separate file (supplemental file) and also cite it in your text.
4. The findings does not show up any part of the qualitative aspect, only tables of qtt have been portrayed in results section. So keeping from the verbatims of the respondents should also be included.
5. Data analysis, further needed to triangulate the qtt with qlt so as to infer scientifically.
6. Some recent literatures for the two areas are recommended:
6.1 For SHI and utilization (pls include in discussion, why quality services affect SHI, what are the alternate strategies for SHI to get success, applicabilty provided to in your case of diabetes and hypertension; from the following, but not limited, (only this as an example) to improve the discussion more argumentative.

Paneru DP, Adhikari C, Poudel S, Adhikari LM, Neupane D, Bajracharya J, Jnawali K, Chapain KP, Paudel N, Baidhya N, Rawal A. Adopting social health insurance in Nepal: A mixed study. Frontiers in Public Health. 2022;10.

6.2. relationship of microfinance status and SHI enrollment: a more and rigorous discussion should be added in discussion section, focusing why less people not on microfinance have not enrolled and relationship of not willing to enroll? This part may be explored from the "financial literacy" or any other theoretical background.
For this, it would be wise to add some literatures from recent and theoretical background.

With regards
Reviewer

***Response****: We are grateful to the Reviewer for the elaborate comments regarding areas of improvement. As previously stated, we have revised the manuscript and left out aspects regarding the qualitative approach since primarily we used a quantitative approach. Furthermore, we have integrated the suggested citation of Paneru et al. (2022) but are unable to examine the relationship between microfinance status and SHI enrolment due to paucity of current knowledge. We feel the latter is, in a way, catered for since among our recommendations we already state “Future studies should seek to gain more in-depth knowledge about microfinance schemes and their potential role as an entry point for health insurance programs for patients in this and other study areas.”*

7. PLOS authors have the option to publish the peer review history of their article ([what does this mean?](https://journals.plos.org/globalpublichealth/s/editorial-and-peer-review-process#loc-peer-review-history)). If published, this will include your full peer review and any attached files.

**Do you want your identity to be public for this peer review?** If you choose “no”, your identity will remain anonymous but your review may still be made public. For information about this choice, including consent withdrawal, please see our [Privacy Policy](https://www.plos.org/privacy-policy).

Reviewer #1: No

Reviewer #3: **Yes:** Chiranjivi Adhikari

***Response****: Thanks.*
